# Supplementary material for: Transcription Factor MAFB as a Prognostic Biomarker for the Lung Adenocarcinoma
Source: Int J Mol Sci. 2022 Sep 1;23(17):9945. doi: 10.3390/ijms23179945 (PMC9456510; doi:10.3390/ijms23179945)
Supplement: Supplementary file 1 [file ijms-23-09945-s001.zip › ijms-1857711-supplementary.pdf]

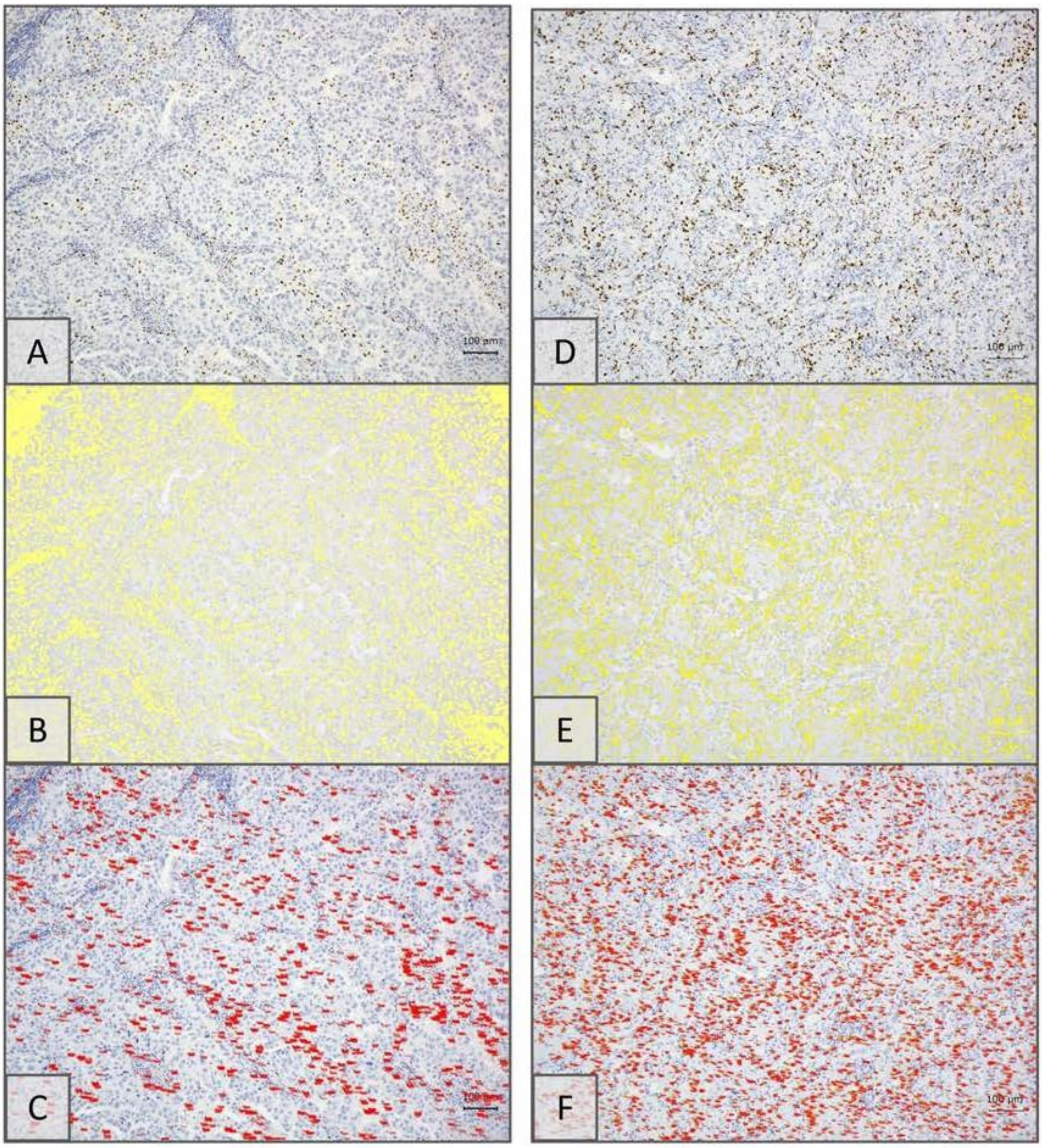

Figure S1. Immunohistochemical analysis of MAFB expression in lung adenocarcinoma showing metastasis potential. The left panel shows (A) Lung adenocarcinoma samples with negative metastasis, (B) the calculation of total tissue area in yellow color, and (C) the number of MAFB positive area in red color. The right panel shows (D) Lung adenocarcinomas samples with positive metastasis, (E) the calculation of total tissue area in yellow color, and (F) the number of MAFB positive area in red color. Scale bar 100 µm, X1 0.

A

B

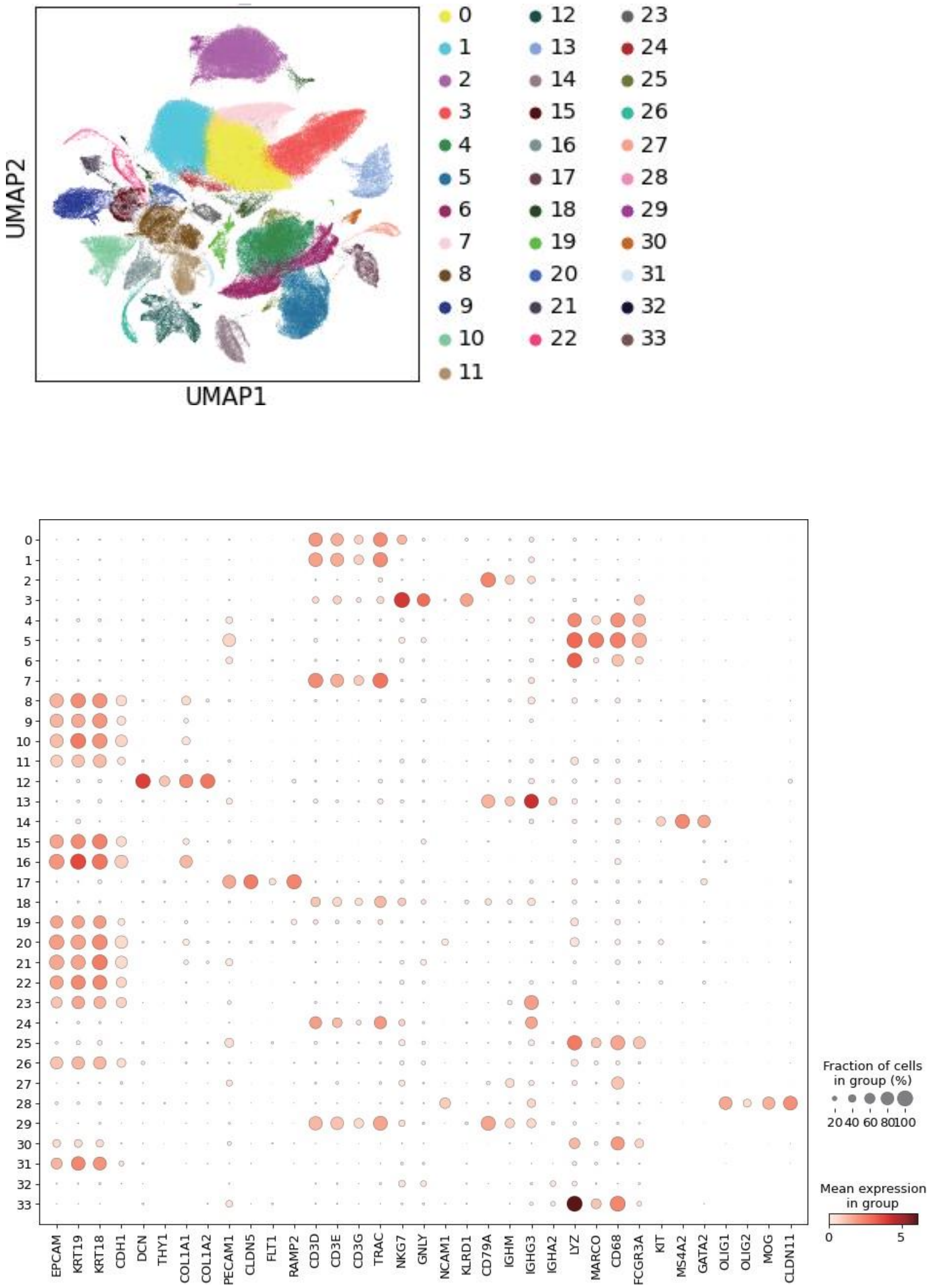

Figure. S2. Clustering strategy of myeloid series. (A) 0-33 clusters were analyzed. (B) myloid series(Cluster 4, 5, 6, 25, 30 and 33) were extrzcted by using myeloid makers, LYZ,MARCO, CD68, FCGR3A.

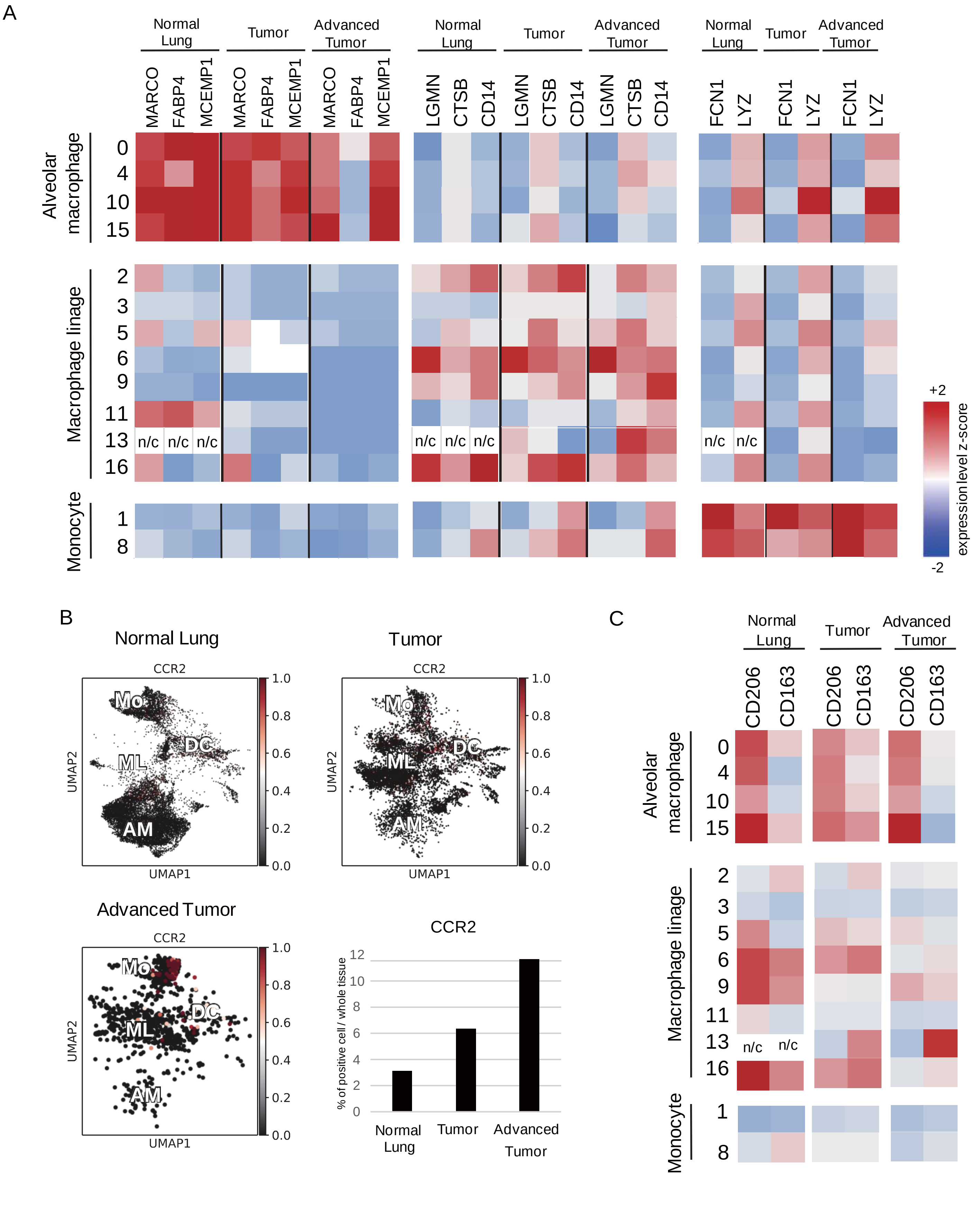

Figure S3. Analysis of myeloid cluster.

(A) Marker gene expression for Alveolar macrophage, Macrophag lineage and Monocyte were shown by heatmap. (B) The CCR2 UMAP of Normal lung, Tumor, Advanced Tumor (C)The other TAM marker, CD206, CD163 expression

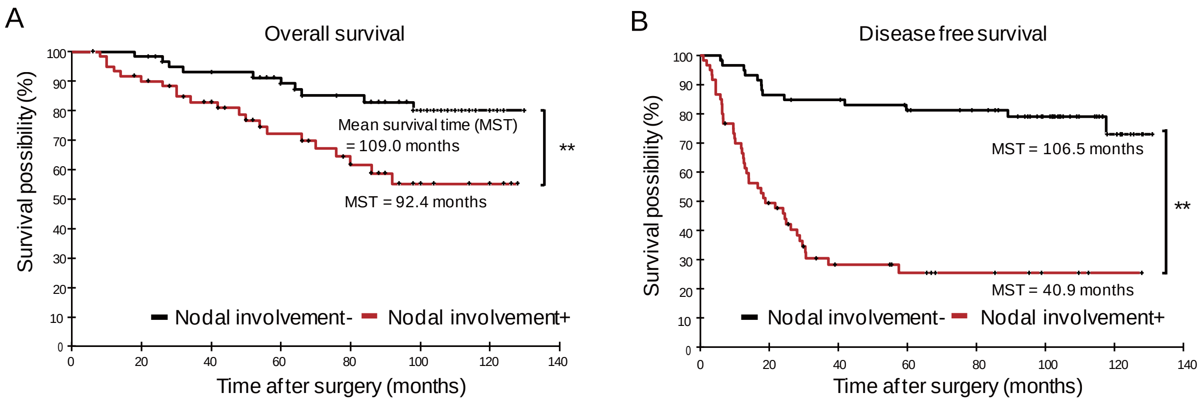

Figure S4. (A) Overall survival of patients with or without nodal involvement. (B)Disease free survival with or without nodal involvement.**,*p*<0.001.

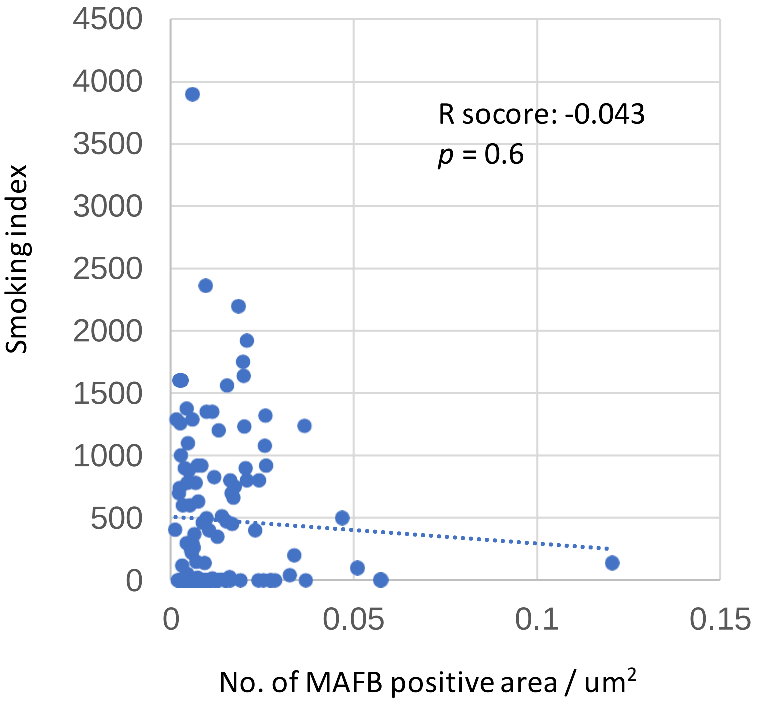

Figure S5. Smoking Index and tumor diameter werenot corelated with MAFB expression Pearson Correlation analysis was performed between MAFBexpression and Smoking index (R socore: -0.043, p = 0.6)

A

Overall survival

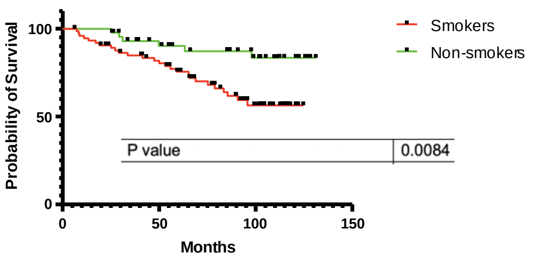

Disease free survival

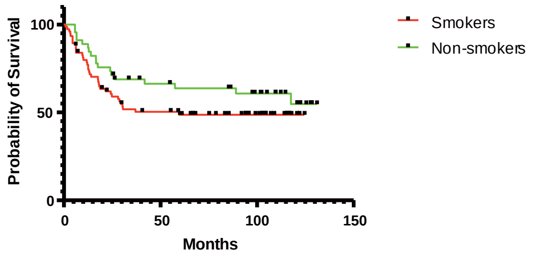

B

ALL female smokers and non-smokers

Overall survival

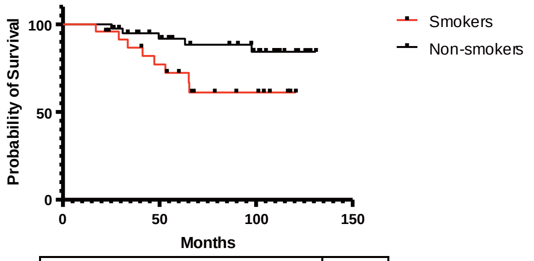

Log-rank (Mantel-Cox) test	
Chi square	4.481
df	1
P value	0.0343
P value summary	*
Are the survival curves sig different?	Yes

ALL female smokers and non-smokers

Disease free survival

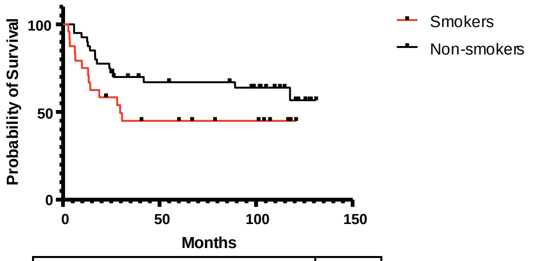

Log-rank (Mantel-Cox) test	
Chi square	2.762
df	1
P value	0.0965
P value summary	ns
Are the survival curves sig different?	No

C

All male smokers and non-smokers

Overall survival

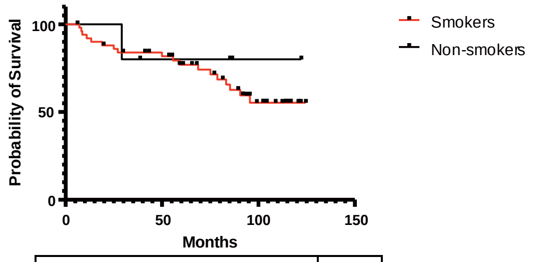

Log-rank (Mantel-Cox) test	
Chi square	0.3803
df	1
P value	0.5374
P value summary	ns
Are the survival curves sig different?	No

All male smokers and non-smokers

Disease free survival

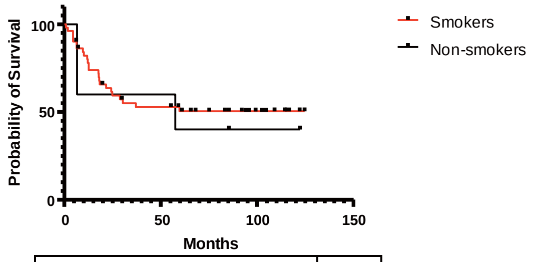

Log-rank (Mantel-Cox) test	
Chi square	0.1741
df	1
P value	0.6765
P value summary	ns
Are the survival curves sig different?	No

Figure S6. (A) Overall survival (OS) and Disease free survival (DFS) of patientsof smokers and non-smokers. (B) OS and DFS of female patiens of smokers and non-smokers. (C) OS and DFS of male patiens of smokers and non-smokers.

Variables	Nodal involovement (N-)<br>(*n* = 60)	Nodal involovement (N+)<br>(*n* = 60)	*P vaule of Fisher's exact test*
**Age (yr)**			
<70	40	34	0.3479
≥70	20	26	
**Gender**			
Male	27	29	0.8549
Female	33	31	
**Smoking history**			
Never	23	22	1
Former or current	37	38	
**Clinical Stage**			
I (*n* = 57)	57	0	**<0.0001**
II+III (*n* = 63)	3	60	
**MAFB positive cells density**			
Low	24	6	**0.0003**
Mid+High	36	54	
**Cancer Recurrence**			
Positive	13	42	**<0.0001**
Negative	47	18	
**Lymphatic permeation**			
Ly(–)	52	19	**<0.0001**
Ly(+)	8	41	
**Vessel invasion**			
V(–)	42	12	**<0.0001**
V(+)	18	48	
**Pleural infiltration**			
PL(–)	43	24	**0.0009**
PL(+)	17	36	

Table S1. Relationship between MAFB+ cells density and the Clinicopathological features in Nodal involovement-negative (*n* = 60) and nodal invlovemnt-positive (*n* = 60) groups inpatients with non-metastatic lung adenocarcinoma. The correlations were evaluated by Fisher's exacttest, *p < 0.05, **p < 0.001
